# Biosynthesis of selenate reductase in *Salmonella enterica*: critical roles for the signal peptide and DmsD

**DOI:** 10.1099/mic.0.000381

**Published:** 2016-12-21

**Authors:** Katherine R. S Connelly, Calum Stevenson, Holger Kneuper, Frank Sargent

**Affiliations:** School of Life Sciences, University of Dundee, Dundee DD1 5EH, Scotland, UK

**Keywords:** *Salmonella enterica*, anaerobic respiration, selenate reductase, protein–protein interactions, twin-arginine signal peptide, system-specific chaperone

## Abstract

*Salmonella enterica* serovar Typhimurium is a Gram-negative bacterium with a flexible respiratory capability. Under anaerobic conditions, *S. enterica* can utilize a range of terminal electron acceptors, including selenate, to sustain respiratory electron transport. The *S. enterica* selenate reductase is a membrane-bound enzyme encoded by the *ynfEFGH-dmsD* operon. The active enzyme is predicted to comprise at least three subunits where YnfE is a molybdenum-containing catalytic subunit. The YnfE protein is synthesized with an N-terminal twin-arginine signal peptide and biosynthesis of the enzyme is coordinated by a signal peptide binding chaperone called DmsD. In this work, the interaction between *S. enterica* DmsD and the YnfE signal peptide has been studied by chemical crosslinking. These experiments were complemented by genetic approaches, which identified the DmsD binding epitope within the YnfE signal peptide. YnfE signal peptide residues L24 and A28 were shown to be important for assembly of an active selenate reductase. Conversely, a random genetic screen identified the DmsD V16 residue as being important for signal peptide recognition and selenate reductase assembly.

## Introduction

*Salmonella enterica* serovar Typhimurium is a Gram-negative γ-Proteobacterium, and an opportunistic animal pathogen, that is able to grow under a variety of environmental conditions (McClelland *et al.*, 2001). As with most facultative anaerobes, *S. enterica* prefers to respire aerobically using short respiratory electron transport chains linked by quinones (Unden *et al.*, 2014). Under anaerobic conditions, however, *S. enterica* can modify its respiratory chain specificity to cope with a range of alternative terminal electron acceptors such as thiosulfate (Stoffels *et al.*, 2012), tetrathionate (Hensel *et al.*, 1999) or nitrate (Lundberg *et al.*, 2004). Indeed, the ability to utilize such a spectrum of electron acceptors has been implicated in the mechanism of infection and pathogenesis used by *S. enterica* (Lopez *et al.*, 2015; Rowley *et al.*, 2012; Winter *et al.*, 2010, 2013).

One unusual terminal electron acceptor that *S. enterica* can utilize is selenate, which is a water-soluble oxidized form of selenium (Köhrl *et al.*, 2000). *S. enterica* can reduce selenate to selenite, which is itself then further reduced to red elemental selenium (Guymer *et al.*, 2009). The selenate reductase is encoded by the *ynfEFGH-dmsD* operon, which is predicted to be transcribed in response to anaerobiosis (Guymer *et al.*, 2009). The YnfE protein is predicted to be the catalytic subunit of the enzyme and is a periplasmically oriented molybdenum-containing enzyme carrying an additional [4Fe–4S] cluster. YnfF is an orthologue of YnfE but does not contribute greatly to cellular selenate reductase activity (Guymer *et al.*, 2009). YnfG is an electron transferring subunit that is predicted to link YnfE to the YnfH quinol oxidase in the inner membrane.

The ultimate step in the biosynthesis of the *S. enterica* selenate reductase is the transmembrane translocation of the YnfE catalytic subunit, most likely in tandem with the electron transferring subunit YnfG (Guymer *et al.*, 2009; Lubitz & Weiner, 2003). YnfE is synthesized as a precursor with an N-terminal twin-arginine signal peptide, which would direct the fully folded protein to the twin-arginine translocation (Tat) machinery for export (Palmer & Berks, 2012). Tat signal peptides share a common structure comprising a polar n-region and a central h-region separated by a conserved SRRxFLK twin-arginine motif (Palmer & Berks, 2012). The h-region is so named because it contains a stretch of 12–20 residues rich in glycine and hydrophobic side chains (Cristóbal *et al.*, 1999). It is common in such complex multi-cofactor, multi-subunit Tat substrates for the cofactor insertion and membrane translocation steps to be coordinated by signal peptide binding proteins (Chan *et al.*, 2014; Sargent, 2007; Turner *et al.*, 2004). The signal peptide binding chaperone that interacts with the selenate reductase (YnfE) signal peptide is called DmsD and it is encoded within the *ynfEFGH-dmsD* operon (Guymer *et al.*, 2009).

*S.* *enterica* DmsD is a member of the TorD/DmsD/NarJ family of peptide binding proteins (Chan *et al.*, 2014). One of the best characterized members of this family is *Escherichia coli* DmsD, which shares 77 % overall sequence identity with *S. enterica* DmsD. *E. coli* DmsD is involved in the assembly of DMSO reductase (Oresnik *et al.*, 2001) and selenate reductase (Guymer *et al.*, 2009) in that organism. Although the *E. coli* DmsD protein can form homo-oligomers *in vitro* (Sarfo *et al.*, 2004), the monomeric from has been structurally characterized (Ramasamy & Clemons, 2009; Stevens *et al.*, 2009, 2012). The *E. coli* DmsD protein is thought to bind to the h-region of the DmsA Tat signal peptide (Shanmugham *et al.*, 2012; Winstone *et al.*, 2013), but a precise binding epitope has not yet been established experimentally.

The *S. enterica* DmsD protein has been structurally characterized and is a 204 amino acid residue monomer consisting of 11 α-helices and no β-strands (Qiu *et al.*, 2008). The purified protein binds tightly to the *S. enterica* YnfE signal peptide (*K*_d_ ~45 nM) (Guymer *et al.*, 2009). In this work, genetic and biochemical approaches were taken to identify precisely the binding epitope for DmsD on the YnfE signal peptide. YnfE residues L24 and A28 were found to be involved in both DmsD recognition and selenate reductase biosynthesis. A YnfE A28Q variant could be rescued by providing DmsD in excess and this was used as a genetic screen to identify inactive variants of *S. enterica* DmsD. One key residue was identified as V16. Finally, a synthetic peptide containing the YnfE binding epitope could be chemically crosslinked to DmsD and signal peptide binding was found to prevent DmsD dimerization *in vitro*.

## Methods

### Growth and construction of *S. enterica* strains.

Naturally attenuated *S. enterica* serovar Typhimurium LT2a was used as the parental strain for this research (Jamieson *et al.*, 1986). *S. enterica* was typically grown in ‘low salt’ LB medium [1 % (w/v) tryptone, 0.5 % (w/v) yeast extract and 0.5 % (w/v) NaCl] and, where necessary, was transformed with plasmids using electroporation. Strains were grown microaerobically with shaking at 37 °C in a volume of 5 ml in a 30 ml universal tube. The Δ*ynfF* strain DIG103 (Guymer *et al.*, 2009) was further modified to separately carry *ynfE* L24Q, *ynfE* A28Q and *ynfE* L33Q alleles (Table 1). Gene replacement on the chromosome was achieved through the use of the vector pMAK705 for homologous recombination (Hamilton *et al.*, 1989). The genetic region ~500 base pairs upstream and downstream of the codons of interest was amplified from DIG103 genomic template using primers 5′-GCGCAAGCTTAGAACATCGTCATTATCACAG-3′ and 5′-GCGCGGATCCGTTGCCGTCGTTGGAACC-3′ and first cloned into pBluescript KS^+^ (Amp^R^) as a *Bam*HI–*Hin*dIII fragment as an intermediate to allow facile codon changes by the QuikChange PCR protocol (Stratagene). Mutant alleles were then transferred to pMAK705 and on to the chromosome of DIG103 (Hamilton *et al.*, 1989) and verified by sequencing.

**Table 1.** Bacterial strains and plasmids used and constructed in this study

	Relevant genotype	Source
**Strains**		
*S. enterica*		
LT2a	Wild strain (attenuated)	Lab. stock
DIG100	As LT2a, Δ*tatABC*	Guymer *et al.* (2009)
DIG103	As LT2a, Δ*ynfF* (STM1498)	Guymer *et al.* (2009)
KM01	As LT2a, Δ*ynfF* *ynfE* L24Q	This work
KM02	As LT2a, Δ*ynfF* *ynfE* A28Q	This work
KM03	As LT2a, Δ*ynfF* *ynfE* L33Q	This work
*E. coli*		
MG1655	F^−^ λ^−^*ilvG*^−^*rfb-50 rph-1*	Blattner *et al.* (1997)
MAE01	As MG1655, Δ*cyaA *::Apra^R^	F. Sargent (unpublished)
**Plasmids**		
pUNI-PROM	As pT7.5, 103 bp *E. coli tat* promoter (Amp^R^)	Jack *et al.* (2004)
pUNI-DmsDst*	As pUNI-PROM, *S. enterica dmsD*	This work
pQE-80L	Overproduction vector (Amp^R^)	Qiagen
pQE-His-TEV-DmsDst	As pQE-80L, *S. enterica dmsD*	This work
pUT18	Encoding adenylate cyclase T18 domain (for N-t fusions), Amp^R^	Karimova *et al.* (1998)
pUT18-spYnfE†	Encoding spYnfE-T18 fusion	This work
pT25	Encoding adenylate cyclase T25 domain (for C-t fusions), Cml^R^	Karimova *et al.* (1998)
pT25-DmsD‡	Encoding T25-DmsD fusion	This work

*Seven derivatives carrying individual codon changes were prepared but not listed here (Fig. 2c).

**Fig. 1.**
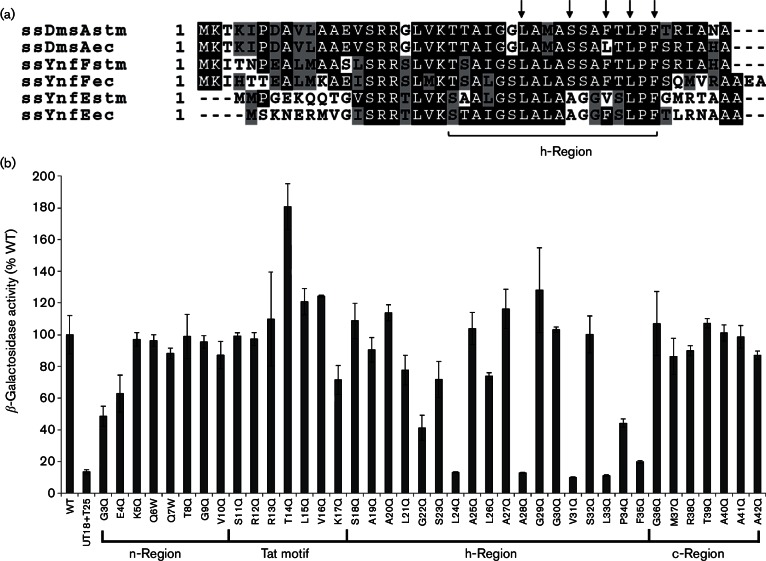
Genetic analysis of the YnfE signal peptide and DmsD interaction. (a) Primary amino acid sequences of the signal sequences of the three binding partners of DmsD from both *E. coli* and *S. enterica* aligned using clustalw (http://embnet.vital-it.ch/software/ClustalW.html), and highlighted using BoxShade (http://embnet.vital-it.ch/software/BOX_form.html). The hydrophobic region of the signal peptides is labelled. Arrows indicate positions of *S. enterica* YnfE L24, A28, V31, L33 and F35. Note that the gene sequence of *S. enterica ynfE* suggests two possible adjacent translation initiation sites. The nomenclature used here assumes that a single methionine is at the N-terminus of YnfE. (b) *E. coli* strain MAE01 (Δ*cyaA *:: Apra) was co-transformed with the vectors pT25-DmsD and pUT18-spYnfE (and mutant versions) before strains were cultured aerobically and β-galactosidase was measured as an indicator of protein–protein interactions. β-Galactosidase activities are displayed relative to native activity generated by the spYnfE–DmsD interaction (WT). Cells containing both empty vectors were used as the negative control (labelled ‘UT18+T25’). In this data set, the positive control (100 %) was recorded as 1646±160 Miller units and the negative control was recorded as 166±30 Miller units. Data expressed relative to the positive control as means±sem (*n*=3).

†Thirty-nine derivatives carrying individual codon changes were prepared but not listed here (Fig. 1b).

‡Seven derivatives carrying individual codon changes were prepared but not listed here (Fig. 3).

### Construction of plasmids for production of DmsD and its variants.

The *S. enterica dmsD* gene was amplified by PCR and cloned as a *Bam*HI–*Pst*I fragment into the pUNI-PROM (Amp^R^) vector, which carries the constitutive *E. coli tat* promoter as an *Eco*RI–*Bam*HI insert (Jack *et al.*, 2004), to give pUNI-DmsDst. This plasmid was further mutagenized using the QuikChange PCR protocol (Stratagene) to yield V16Q, W91Q, R94Q, S96Q, G100Q, T103Q and G171Q derivatives. For high-level overexpression of *dmsD* in *E. coli*, the *S. enterica dmsD* gene was amplified with primers 5′-GCGCGGATCCGAAAACCT GTATTTTCAGGGCATGACCACTTTTTTACAACGTGATG-3′, which would incorporate an in-frame tobacco etch virus (TEV) protease cleavage site sequence, and 5′-GCGCAAGCTTTTATTAACGGAATAACGGTTTTACAGCG-3′ before being cloned as a *Bam*HI–*Hin*dIII fragment into pQE-80L (Qiagen).

### Bacterial two-hybrid analysis.

The bacterial two-hybrid system is based on the reconstitution of an adenylate cyclase signal transduction pathway in an *E. coli cyaA* mutant where two complementary fragments, T18 and T25, of a soluble adenylate cyclase domain from *Bordetella pertussis* toxin are fused to two potentially interacting proteins (Karimova *et al.*, 1998). The *S. enterica dmsD* gene was incorporated in-frame at the 3′ end of the T25 coding sequence on pT25 (Cml^R^). This plasmid was further mutagenized to yield V16Q, W91Q, R94Q, S96Q, G100Q, T103Q and G171Q versions. A fragment of DNA encoding the YnfE Tat signal peptide was amplified by PCR and cloned in-frame at the 5′ end of the T18-encoding sequence on plasmid pUT18 (Amp^R^). The resultant pUT18-spYnfE vector was mutagenized a further 39 times to provide a comprehensive bank of site-directed mutants covering every codon of the signal peptide. To assay for an interaction, chemically competent *E. coli* MAE01 (as MG1655 Δ*cyaA*) cells were transformed with relevant vectors and plated onto MacConkey agar plates containing 1 % (w/v) maltose as the carbon source. Plates were incubated for 2 days at 30 °C before inoculation of 5 ml LB medium and grown at 30 °C overnight with 200 r.p.m. shaking. These cultures were then sub-cultured and grown under the same conditions until an approximate OD_600 nm_ of 0.5 was reached. The cellular β-galactosidase activity was then measured (Karimova *et al.*, 1998).

### Random mutagenesis of *S. enterica dmsD*.

Random mutagenesis was carried out according to a PCR mutagenesis procedure described by Vartanian *et al.* (1996). The *dmsD* gene was amplified from plasmid pUNI-DmsDst in the presence of 0.5 mM MnCl_2_ and strong dNTP bias (dTTP and dGTP at a concentration of 1000 µM each versus dATP and dCTP at 75 µM) using primers yigR1634 (5′-GCTGATTTTTTCATCGCTCAAG-3′) and T7.5 rev (5′-CGCTGAGATAGGTGCC-3′). The resulting PCR product was purified, restricted with *Bam*HI and *Hin*dIII and cloned into pUNI-PROM. Recombinant plasmids were used to transform *E. coli* XL10-Gold cells (Stratagene) and 10 random clones were sequenced to estimate the average error rate, which was ~1.5 % (4–18 errors per 615 bp *dmsD*). Remaining colonies (approximately 300 000) were suspended in LB medium and used to inoculate a 200 ml culture supplemented with ampicillin to a starting OD_600 nm_ of 0.2. Cells were grown aerobically to a final OD_600 nm_ of ~2 and plasmid DNA was isolated from 20 % of the culture volume and stocked as the *dmsD* mutant library.

### Purification of affinity-tagged DmsD.

Proteins were overproduced from pQE80L-based vectors following transformation of *E. coli* BL21 (DE3) pLysS. A single colony was used to inoculate a 5 ml overnight starter culture in LB plus appropriate antibiotics before 500 ml cultures were then inoculated at 1 : 1000 dilution. Cultures were grown aerobically in 2 litre baffled flasks at 37 °C with 170 r.p.m. shaking until an OD_600 nm_ of 0.6 was reached. Protein production was initiated by addition of 2 mM IPTG (Sigma-Aldrich). Cultures were then incubated overnight at 18 °C before cells were harvested by centrifugation. Cell pellets were suspended in 25 mM Tris/HCl (pH 7.5), 25 mM imidazole and 250 mM NaCl at 10 ml g^−1^ cells (wet weight). Protease inhibitors (protease inhibitor cocktail set III, EDTA-free; Calbiochem), lysozyme and DNase were added before cells were lysed by three times passage at 15 000 p.s.i. through an Emulsiflex C3 high-pressure homogenizer. Cell debris was removed by centrifugation at 27 143*** g*** at 4 °C. The resultant supernatant was filtered through a 0.45 µM membrane filter before being loaded onto a 5 ml HisTrap HP column (GE Healthcare) pre-equilibrated with 25 mM Tris/HCl (pH 7.5), 25 mM imidazole and 250 mM NaCl at 0.5 ml min^−1^. Bound protein was eluted in the same buffer with an imidazole gradient of 25–500 mM imidazole over five column volumes. Fractions containing the protein of interest were identified by SDS-PAGE and were pooled and concentrated if required. To remove the affinity tag, purified protein was mixed with in-house TEV*^His^* protease in a ratio of 10 : 1 (per milligram). This was dialysed for 16 h at 4 °C against 25 mM Tris/HCl (pH 7.5), 25 mM imidazole, 250 mM NaCl and 1 mM DTT. Cleaved DmsD protein was isolated by reverse immobilized metal affinity chromatography with the unbound fraction retained for further experimentation.

### Chemical crosslinking *in vitro*.

DmsD and its variants were dialysed into 50 mM HEPES (pH 7.6) and 150 mM KCl ‘crosslinking buffer’. Synthetic peptides were suspended in the same buffer to a concentration of 2 mM. Reactions were carried out in 100 µl. Disuccinimidyl suberate (DSS) was added to the reaction to a final concentration of 1 mM, 1-ethyl-3-(3-dimethylaminopropyl)carbodiimide hydrochloride (EDC) to 2 mM and formaldehyde to 1 % (v/v) before subsequent 30 min incubation on ice. Reactions were stopped by the addition of 10 mM Tris/HCl (pH 8.0) before being mixed in a 1 : 1 ratio with Laemmli disaggregation buffer (Laemmli, 1970). Synthetic peptides of various sequence stretches (Table 2) were synthesized by Severn Biotech (Kidderminster) to the highest degree of purity and do not contain N-terminal amine or C-terminal carboxyl groups.

**Table 2.** Synthetic peptides used in this study

Name	Amino acid sequence	Length (aa)	Molecular mass (kDa)	Use
Peptide 1	SLALAAGGVSLPFGMRK	17	1.675	Crosslinking
Peptide 2	SRRTLVKSAALGSLALAAGGVSLPFGMRK	29	2.915	Crosslinking
Peptide 3	SRRTLVKSAALGSLALAAGGVSLPFGMR_	28	2.787	Crosslinking

### Protein analytical techniques.

Proteins were analysed by SDS-PAGE by the method of Laemmli (1970). Where stated, Western immunoblotting was carried out by transferring proteins previously separated by SDS-PAGE onto nitrocellulose (Dunn, 1986). Secondary antibodies conjugated to horseradish peroxidase (Bio-Rad) were used allowing development of immunoblots by chemiluminescence.

## Results

### Genetic evidence for the DmsD binding epitope on the *S. enterica* YnfE signal peptide

In order to understand further the role of the YnfE signal peptide in selenate reductase activity, the first step was to identify the binding epitope for DmsD on the signal peptide. To do this, a glutamine scanning mutagenesis approach was taken combined with a bacterial two-hybrid genetic screen based on reconstitution of adenylate cyclase activity. When the T18 and T25 fragments of *B. pertussis* toxin adenylate cyclase domain are co-expressed as separate polypeptides, they do not interact. However, adenylate cyclase activity can be restored to an *E. coli* Δ*cyaA* host strain if T18 and T25 are brought close together by fusing each to interacting proteins (Karimova *et al.*, 1998).

In this work, the 42-residue YnfE Tat signal peptide (spYnfE) was placed at the N-terminus of T18. Next, a plasmid was constructed that encoded a T25-DmsD fusion protein, with *S. enterica* DmsD covalently attached to the C-terminus of T25. Co-expression of both plasmids in the appropriate reporter stain demonstrated a strong interaction between spYnfE and DmsD (Fig. 1). Scanning mutagenesis of the YnfE sequence was then performed, involving the substitution of each individual amino acid (from E3 to A42, but excluding Q6 and Q7) of the signal peptide with glutamine. Glutamine is a polar amino acid rarely present in Tat signal peptides (Cristóbal *et al.*, 1999), and therefore is an ideal amino acid for attempting to disrupt Tat signal peptide function. In the case of YnfE Q6 and Q7, these side chains were substituted with tryptophan.

Using the site-directed mutagenesis approach, the amino acids of the highly conserved twin-arginine motif were found to be not involved in chaperone binding in this assay (Fig. 1). This was also the case regarding the entire n-region of the YnfE signal peptide (Fig. 1). Similarly, the polar c-region of the signal peptide was found to be not critical for the interaction (Fig. 1). However, the data clearly highlighted the hydrophobic stretch of the signal peptide as important in interactions with DmsD (Fig. 1). The spYnfE L24Q, A28Q, V31Q, L33Q and F35Q variants all displayed reduced levels of β-galactosidase activity – approximately 10 % of that observed for the native signal peptide (Fig. 1), which were in fact similar values to the background levels observed for the negative controls (Fig. 1).

### Signal peptide amino acid substitutions affect selenate reduction *in vivo*

Bacterial two-hybrid analysis implicated five YnfE residues L23, A28, V31, L33 and F35 as possibly playing a role in DmsD interactions. To explore the physiological roles of these YnfE residues, the next task was to incorporate the mutant alleles onto the native copy of *ynfE* on the *S. enterica* chromosome. To do this, strain DIG103, which contains an in-frame deletion of the gene coding for YnfF, which shares 71 % sequence overall identity with YnfE, was selected since this strain produces only YnfE as its sole selenate reductase catalytic subunit (Guymer *et al.*, 2009). Using technology based on homologous recombination, it was possible to construct three mutant strains (Table 1). These were KM01 (Δ*ynfF*, *ynfE* L24Q), KM02 (Δ*ynfF*, *ynfE* A28Q) and KM03 (Δ*ynfF*, *ynfE* L33Q) (Table 1).

**Fig. 2.**
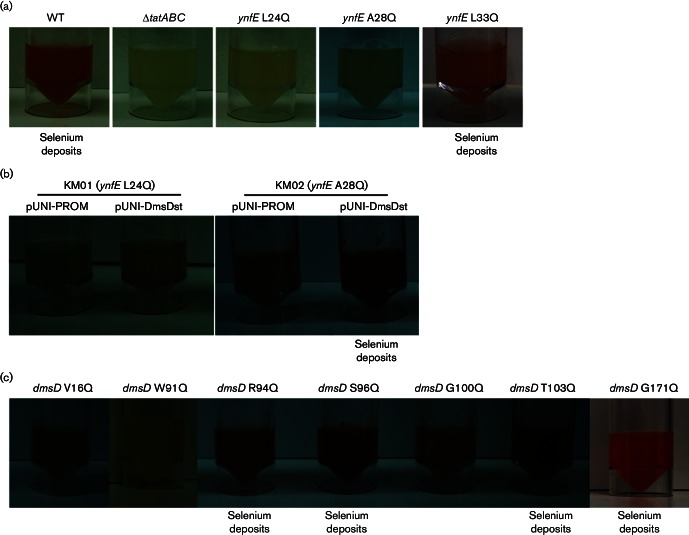
Identification of YnfE signal peptide and DmsD residues required for *in vivo* selenate reduction. (a) *S. enterica* strains DIG103 (ΔSTM1498, labelled ‘WT’), DIG100 (Δ*tatABC*), KM01 (*ynfE* L24Q), KM02 (*ynfE* A28Q) and KM03 (*ynfE* L33Q) were grown overnight under microaerobic conditions in LB+10 mM sodium selenate. (b) Strains KM01 (*ynfE* L24Q) and KM02 (*ynfE* A28Q) were transformed with either empty pUNI-PROM or pUNI-DmsDst. A resultant colony was then grown overnight in microaerobic liquid LB culture containing 10 mM sodium selenate. (c) *S. enterica* strain KM02 (*ynfE* A28Q) was transformed with pUNI-DmsDst single mutants encoding the substitutions indicated. Single colonies were inoculated into LB medium containing 10 mM sodium selenate before being grown overnight in microaerobic conditions. In all cases, activity of selenate reductase is visible through production of red deposits in the cultures.

A direct biochemical assay for selenate reductase activity has not been developed for the *S. enterica* enzymes, most likely because of poor expression levels and poor reactivity with redox dyes (Guymer *et al.*, 2009); however, it is possible to detect selenate reduction by a facile growth test (Guymer *et al.*, 2009). Incubation of cells in the presence of 10 mM selenate leads to initial reduction of selenate to selenite by selenate reductase and then further reduction of selenite to a red elemental selenium precipitate. In this case, the three mutant strains KM01 (*ynfE* L24Q), KM02 (*ynfE* A28Q) and KM03 (*ynfE* L33Q) were cultured in rich medium containing selenate (Fig. 2a). Strains producing the spYnfE variants L24Q and A28Q were clearly devoid of selenate reductase activity (Fig. 2a), while the spYnfE variant L33Q retained the ability to reduce selenate *in vivo* (Fig. 2a).

### Overproduction of DmsD rescues selenate reductase activity in the YnfE A28Q variant

The implication of spYnfE L24 and A28 in DmsD binding (Fig. 1), together with the selenate reductase-minus phenotypes of KM01 (spYnfE L24Q) and KM02 (spYnfE A28Q) (Fig. 2), immediately suggested the possibility of designing a second-site suppressor positive screen. This would involve constructing a random mutant library for *dmsD* that could be screened for those able to recognize the variant signal peptides and rescue selenate reductase activity. To begin to explore this idea, the KM01 (spYnfE L24Q) and KM02 (spYnfE A28Q) strains were transformed with pUNI-DmsDst, encoding native *S. enterica* DmsD, and the empty vector pUNI-PROM. Following overnight growth with sodium selenate, KM01 (spYnfE L24Q) containing either pUNI-PROM or pUNI-DmsDst remained devoid of selenate reductase activity (Fig. 2b). However, it was clear that the KM02 (spYnfE A28Q) with pUNI-DmsDst was restored for *in vivo* selenate reductase activity (Fig. 2b).

The KM01 (spYnfE L24Q) strain was deemed suitable for screening a *dmsD* mutant library. This was prepared by error-prone PCR where the final resultant error rate was 1.5 % (4–18 errors across 615 bp of *dmsD*). The PCR products were cloned into vector pUNI-PROM resulting in around 300 000 individual clones. Screening the library involved transformation of KM01 (spYnfE L24Q) and plating directly onto LB agar plates containing 10 mM sodium selenate and appropriate antibiotic. The plates were then incubated for 36 h under anaerobic conditions with colonies then being picked for further analysis according to colour. For KM01 (spYnfE L24Q), red colonies were desired as this would indicate reconstitution of selenate reductase activity. Unfortunately, no selenate reductase-positive clones were identified in this screen.

### A negative screen for isolation of DmsD variants affecting selenate reductase activity

The colorimetric plate test was next adapted for the isolation of inactive variants of *S. enterica* DmsD. In this case, the behaviour of KM02 (spYnfE A28Q) when transformed with pUNI-DmsDst was exploited (Fig. 2b). The hypothesis to be tested was that since the combination of the YnfE A28Q substitution and increased levels of active DmsD allowed red selenium deposits to be observed, then this assay may also be used to select for inactive variants of DmsD that do not induce selenium precipitation.

The *dmsD* mutant library was used in the transformation of KM02 (spYnfE A28Q), with cells plated directly onto LB (ampicillin) agar. Individual colonies were then picked onto fresh solid medium plates containing 10 mM sodium selenate and appropriate antibiotic. The plates were then incubated for 36 h under anaerobic conditions and colonies that remained white (and so contained no selenate reductase activity) were used to inoculate 30 ml anaerobic liquid cultures. Aliquots of cells were then harvested, whole cell proteins separated by SDS-PAGE and Western immunoblotting carried out with an anti-DmsD (*E. coli*) serum to screen for clones producing full-length DmsD (Fig. S1, available in the online Supplementary Material).

This approach identified three clones that produced full-length recombinant DmsD but were unable to rescue selenate reductase activity in KM02 (spYnfE A28Q). Gene sequencing of the three clones revealed that one encoded a single W91R substitution in DmsD, one carried two mutations encoding R94H and G100S and the third contained four mutations coding for a DmsD variant with V16G, S96G, T103I and G171R substitutions. Next, each of the seven identified residues was individually targeted and a bank of seven vectors was prepared, encoding variant DmsD proteins carrying glutamine substitutions at positions 16, 91, 94, 96, 100, 103 and 171. The KM02 (spYnfE A28Q) strain was transformed individually by each of the seven plasmids and cultured in the presence of 10 mM selenate (Fig. 2c). It is clear that V16Q, W91Q and G100Q versions of DmsD are unable to coordinate selenate reductase biosynthesis in *S. enterica* (Fig. 2c).

**Fig. 3.**
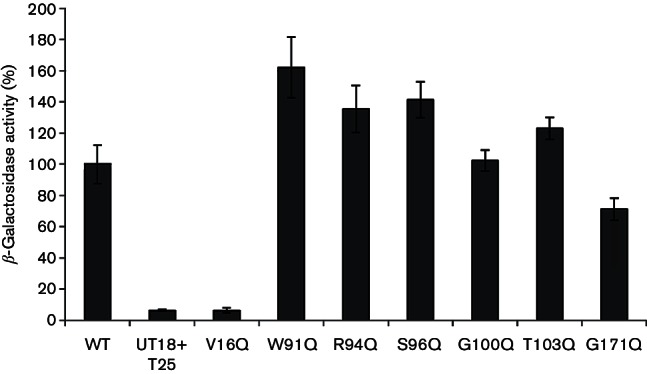
DmsD variant V16Q is unable to recognize the YnfE signal peptide. *E. coli* strain MAE01 (Δ*cyaA *::Apra) was co-transformed with vectors pUT18-spYnfE and pT25-DmsD plus variants and β-galactosidase was measured as an indicator of protein–protein interactions. β-Galactosidase activities are displayed relative to native spYnfE–DmsD activity (WT). Cells containing both empty vectors were used as the negative control (UT18+T25). In this data set, the positive control (100 %) was recorded as 1718±212 Miller units and the negative control was recorded as 108±10 Miller units. Data expressed relative to the positive control as means±sem (*n*=3).

For quantification of the ability of the identified DmsD variants to interact with the signal sequence of YnfE, the bacterial two-hybrid system was utilized (Fig. 3). In this experiment, the seven *dmsD* mutations identified in the random screen were moved on to plasmid pT25-DmsD. Interactions of these DmsD variants were tested against the spYnfE-T18 fusion through use of the β-galactosidase reporter system (Fig. 3). The results show that DmsD W91Q, R94Q, S96Q, G100Q, T103Q and G171Q typically displayed β-galactosidase activity levels close to or in excess of native levels (Fig. 3). These were therefore considered to be essentially unimpaired in signal peptide recognition. However, the DmsD V16Q variant clearly differed in its β-galactosidase activity, with levels almost identical to that of the empty vector negative control (Fig. 3).

Taken altogether, these experiments suggest that YnfE signal peptide residue A28 is specifically involved in the DmsD interaction as part of a minimum binding epitope covering the C-terminal half of the h-region. The YnfE A28Q variant cannot be impaired in Tat transport per se, since it can be rescued by simply increasing cellular levels of DmsD. In addition, these experiments identified DmsD V16 as playing a critical role in the interaction with the YnfE signal peptide and, interestingly, two other DmsD variants (W91Q and G100Q) were isolated that retain signal peptide recognition capabilities but are unable to restore selenate reductase activity to a mutant strain.

### Using chemical crosslinking to study the spYnfE–DmsD interaction

The genetic experiments (Figs 1 and 2) have helped us to identify the potential binding epitope for DmsD on the YnfE signal peptide. The 16 residue sequences between YnfE S23 and R38 contain all of the critical amino acids for DmsD binding (Fig. 1). In order to biochemically characterize binding by this epitope, with the long-term view of obtaining structural information on the binary complex, synthetic peptides were synthesized covering this region (Table 2). These synthetic peptides do not contain terminal amino or carboxyl groups; however, because the genetic data described here point to no role for the Tat motif and a binding epitope at the extreme C-terminus of the YnfE signal, an extra lysine residue was incorporated in the peptide sequences at the C-terminal end to aid crosslinking experiments (Table 2).

**Fig. 4.**
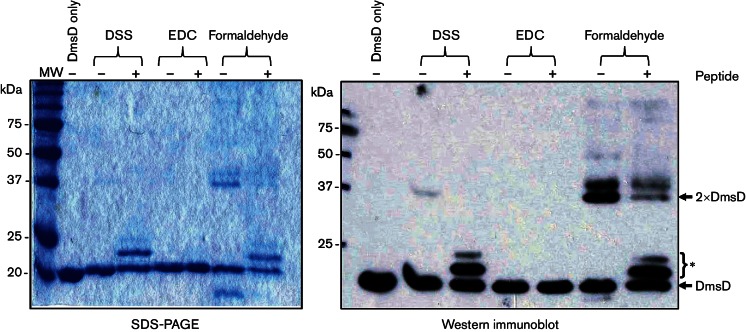
DSS or formaldehyde, but not EDC, can crosslink a peptide ligand to DmsD. Purified DmsD (2 µM) and synthetic peptide 2 (Table 2; 100 µM) were incubated together in a final volume of 100 µl prior to the addition of chemical crosslinkers [1 mM DSS; 1 mM EDC; 1 % (v/v) formaldehyde, final concentrations]. Reactions were stopped after 30 min with 50 mM Tris/HCl (pH 8.0) before 10 µl samples were analysed by SDS-PAGE [17 % (w/v) acrylamide gel] and Western immunoblotting. Antibodies used were anti-*E. coli* DmsD (1 : 20 000) with a horseradish peroxidase-conjugated anti-rabbit IgG secondary (1 : 10 000). The asterisk marks the position of peptide crosslinked DmsD.

Recombinant *S. enterica* DmsD was produced in *E. coli*, purified and its N-terminal hexa-histidine tag removed by proteolysis (Fig. S2). The next step was to experiment with chemical crosslinking of a synthetic signal peptide to DmsD (Fig. 4). Purified DmsD and the synthetic 29-mer peptide (peptide 2, Table 2), which contains an additional lysine at the C-terminus, were incubated together in solution and three different chemical crosslinkers were tested for their ability to induce crosslinks (Fig. 4). DSS is a chemical crosslinker that is able to covalently link lysine side chains and other primary amine groups that are within 11.4 Å. EDC is a zero-length crosslinker capable of inducing crosslinks between carboxyl groups and primary amines, and formaldehyde is a bifunctional crosslinker that can be used to form covalent bonds over distances of approximately 2.3 Å, which primarily reacts with lysine and tryptophan side chains as well as primary amines at N-termini (Sutherland *et al.*, 2008). Analysis of DmsD samples by SDS-PAGE and Western immunoblotting after DSS treatment revealed an additional two slower-migrating bands in the presence of peptide 2 (Fig. 4). This indicated that DSS was probably able to form a covalent crosslink between DmsD and the synthetic peptide 2 (Fig. 4). In comparison, samples treated with EDC did not show any additional banding patterns in the presence of peptide 2 (Fig. 4). This suggests that EDC is not a suitable crosslinker for such experiments. It should be noted, however, that it is recommended to include *N*-hydroxysuccinimide, or its water-soluble analogue sulfo-*N*-hydroxysuccinimide, in reactions with EDC to improve efficiency of the reaction. This was not done in these experiments. In contrast, formaldehyde was able to induce crosslinking (Fig. 4). In this case, it was notable that the sample of DmsD without additional peptide produced a species close to ~40 kDa, the predicted size for DmsD dimer (Fig. 4). Very interestingly, upon the addition of peptide 2 to the formaldehyde experiment, the amount of DmsD dimer noticeably decreased and additional banding appeared similar to the apparent DmsD peptide mass observed with DSS (Fig. 4).

**Fig. 5.**
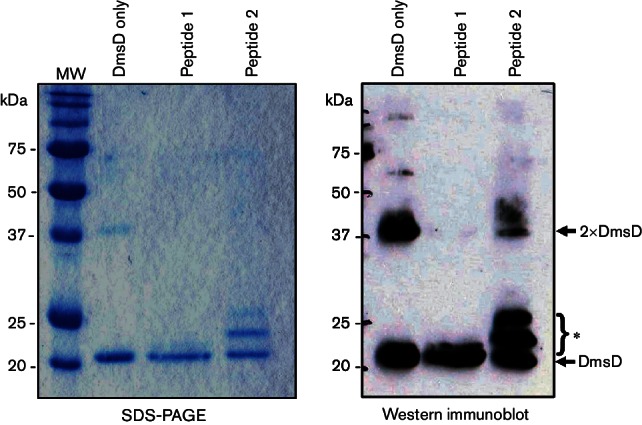
DSS-mediated crosslinking of signal peptides to DmsD prevents homo-dimerization of the chaperone. purified DmsD (2 µM) and synthetic peptides 1 and 2 (Table 2; 100 µM) were incubated together with 1 mM DSS (final concentration) in a final volume of 100 µl. The reaction was stopped after 30 min with the addition of 50 mM Tris/HCl (pH 8) before 10 µl samples were analysed by SDS-PAGE [17 % (w/v) acrylamide gel] and Western immunoblotting. Antibodies used were anti-DmsD (1 : 20 000) and an anti-rabbit IgG secondary (1 : 10 000). The positions of the DmsD monomer (DmsD), dimer (2×DmsD) and peptide crosslinked forms (*) are indicated.

Since crosslinking could be induced using amine-specific DSS and peptide 2 (Table 2, Fig. 4), the interaction between DmsD and the minimal predicted binding epitope on YnfE was tested. Peptide 1 was a 17-mer and contained just one non-native lysine at the C-terminal end (Table 2). Incubation of DmsD with peptide 1 and DSS resulted in a small molecular mass shift indicative of peptide attachment (Fig. 5). Moreover, in this experiment, the observation of the DmsD dimer was again observed when no peptide was added (Fig. 5) and incubation with peptide 1 completely prevented self-self crosslinking by DmsD (Fig. 5).

**Fig. 6.**
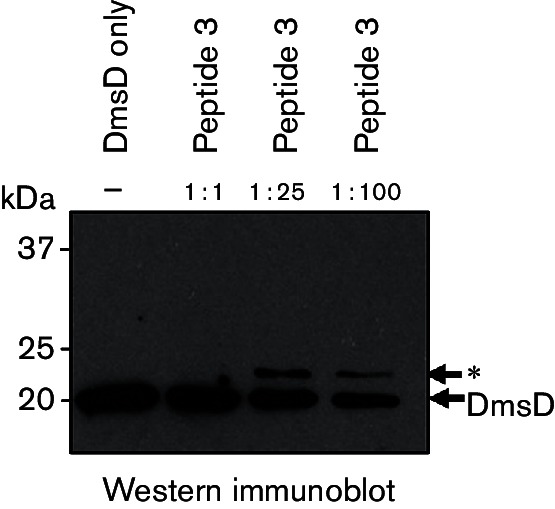
A synthetic peptide with a single internal lysine within the Tat motif can only be weakly crosslinked to DmsD. DSS crosslinking was induced between purified DmsD and synthetic peptide 3 (Table 2), which is a 28-mer carrying a single lysine within the twin-arginine motif. Varying molar ratios of DmsD (1 µM) to peptide (as indicated) were incubated for 1 h with 1 mM DSS. The sample marked (−) contains no peptide and no crosslinker. Reactions were stopped by the addition of 50 mM Tris/HCl (pH 8), before 10 µl samples were analysed by SDS-PAGE [17 % (w/v) acrylamide gel] and Western immunoblot (anti-DmsD, 1 : 20 000; anti-rabbit IgG secondary, 1 : 10 000). The asterisk marks the position of peptide crosslinked DmsD.

Given that peptide 2 contains two lysine residues (Table 2), but peptide 1 can still crosslink to DmsD (Fig. 5), it could be inferred that the C-terminal engineered lysine residue is important for the crosslink to form. To test this experimentally, a third synthetic peptide (peptide 3) was synthesized that contained a single internal lysine close to the twin-arginine motif (Table 2). When Western immunoblotting was used, crosslinked complexes between peptide 3 and DmsD could be weakly detected (Fig. 6); however, the efficiency of crosslinking was clearly weaker in comparison with experiments using peptide 2 (Figs 1 and 5) with incubation in 100× peptide 3 producing only a weak complex signal (Fig. 6). Taken together, these data indicate that all three peptides tested can form interactions with DmsD to differing extents *in vitro* and the binary complex can be chemically crosslinked using DSS.

## Discussion

### A semi-conserved DmsD binding epitope on Tat signal peptides

The *S. enterica* DmsD protein binds directly to at least three Tat signal peptides in the cell, including those of DmsA, YnfE and YnfF (Guymer *et al.*, 2009). These three Tat signal peptides already contain obvious stretches of sequence conservation (Fig. 1a). However, the n-region of the YnfE signal peptide, which precedes the conserved Tat motif, is very different in primary structure from that of DmsA and YnfF (Fig. 1a). This immediately suggests that, if the DmsD recognition sequence is to be similar among the three signal peptides, the n-region is not involved in chaperone binding. The genetic data presented here appear to corroborate that hypothesis, with signal peptide–DmsD interactions recorded for all variants constructed between YnfE G3 and V10 (Fig. 1a).

These data also point to no role for the twin-arginine motif in binding *S. enterica* YnfE by DmsD (Fig. 1a). In this regard, the behaviour of *S. enterica* DmsD is similar to *E. coli* TorD, which does not rely on the Tat motif for signal peptide recognition (Buchanan *et al.*, 2008). Note, however, that, for *Archaeoglobus fulgidus* TtrD, which is related to DmsD, and *E. coli* NapD, which is from a different protein family to DmsD (Turner *et al.*, 2004), the twin-arginine motif, or residues close to it, was found to be part of the chaperone binding epitope (Coulthurst *et al.*, 2012; Grahl *et al.*, 2012). There is also some biochemical evidence that *E. coli* DmsD does interact with the twin-arginine motif to some extent (Winstone & Turner, 2015), while the hydrophobic h-region contains the major drivers for specificity (Shanmugham *et al.*, 2012; Winstone *et al.*, 2013).

In this work, certain key side chains in the YnfE signal peptide h-region were found to be critical for observation of a clear interaction with *S. enterica* DmsD *in vivo* (Fig. 1b). The bacterial two-hybrid data suggested that five residues could be important – YnfE L24, A28, V31, L33 and F35 (Fig. 1b). Of these, the A28 residue was established here as being important for DmsD recognition and biosynthesis of selenate reductase. Based on the two-hybrid data, three mutant strains were constructed encoding YnfE L24Q, YnfE A28Q and YnfE L33Q. Although L33 is completely conserved in all target peptides for DmsD (this is equivalent to L37 in *E. coli* DmsA), a strain carrying an L33Q mutation in the *ynfE* gene was still able to correctly assemble selenate reductase (Fig. 2a). On the other hand, strains producing YnfE L24Q or A28Q were devoid of selenate reductase activity. The equivalent of L24 is completely conserved in all DmsD target peptides (Fig. 1a); however, it is most likely that this residue has a dual role in both DmsD binding and Tat translocation. This is because no suppressors whatsoever were found in the *dmsD* mutant library screen. The YnfE A28Q variant, however, could be rescued by supplying extra native DmsD to the cell, which would be expected if the YnfE A28Q substitution reduced the affinity of the signal peptide for DmsD but was not important for final Tat translocation following enzyme assembly. Interestingly, the tolerance of the YnfE signal peptide to an A28Q substitution may have been predictable. In other DmsD-dependent systems, this residue is often a polar serine side chain (Fig. 1a).

Overall, this section of work identified a short stretch of hydrophobic residues between YnfE L24 and F35 as being the location of the DmsD binding epitope. When plotted on a helical wheel, the L24, A28, V31 and F35 side chains align perfectly onto one face of the α-helix (Fig. S3). Interestingly, L33 is an outlier, being predicted to locate on the opposite side of the helix to the L24/A28/V31/F35 face (Fig. S3). This, taken together with the positive selenate reductase activity in a YnfE L33Q strain, may suggest that the L33Q result in the bacterial two-hybrid study was a ‘false negative’. The data uncover a semi-conserved DmsD binding site at the C-terminal end of the signal peptide h-region for YnfE, YnfF and DmsA.

### Signal peptide binding by DmsD

The second strand of this study involved the biochemical properties of DmsD itself. A random genetic screen identified three residues that may be critical for the function of *S. enterica* DmsD. The first of these is DmsD V16, which appears to be important for signal peptide binding but has not been implicated previously in other studies of chaperone function. The *S. enterica* DmsD V16 residue lies close to G18 of *E. coli* DmsD implicated in peptide binding by NMR (Stevens *et al.*, 2012). The other interesting DmsD variants identified in this work (W91Q and G100Q) highlight the possibility that DmsD may play a biochemical role in the cell beyond peptide recognition and binding. Both *S. enterica* DmsD variants were capable of peptide binding using a genetic test, but were impaired in assembly of an active selenate reductase.

The *E. coli* DmsD protein has been extensively mutagenized and peptide binding of the variant proteins analysed *in vitro* (Chan *et al.*, 2008; Winstone & Turner, 2015). A key residue for efficient binding of the *E. coli* DmsA signal peptide by *E. coli* DmsD is W87, which has a direct equivalent in *S. enterica* DmsD and is distinct from W91 identified here (Winstone & Turner, 2015). While the physiological function of *E. coli* DmsD W87S and W87Y variants has not been reported, the identification of an *S. enterica* variant in this region by a completely random screen reinforces the critically important nature of this part of DmsD to its function.

### Signal peptide binding prevents dimerization of DmsD *in vitro*

The chemical crosslinking experiments described here introduce a hitherto unexplored approach to studying signal peptide binding by Tat proofreading chaperones. The main conclusions to be drawn are that amine-specific crosslinkers such as DSS and formaldehyde are an appropriate choice for studying chaperone–Tat signal peptide interactions, and that the self-dimerization of DmsD frequently observed *in vitro* can be impaired by peptide binding. The latter was the most surprising observation from the crosslinking experiments. DmsD-like proteins are known to form dimers and higher oligomers *in vitro* (Guymer *et al.*, 2010; Sarfo *et al.*, 2004; Tranier *et al.*, 2002). In this work, dimer formation can be captured by the addition of DSS or formaldehyde as a crosslinker (Figs 4 and 5). Most interestingly, however, the formation of DmsD dimer can be prevented by co-incubation with signal peptide (Figs 4 and 5). This suggests that the dimerization interface comprises, or is close to, the signal peptide binding site on the surface of DmsD.

Another interesting observation was the possible generation of two different DmsD–peptide crosslinked species under some circumstances (Figs 4 and 5). By Western immunoblotting, a clearly slower-migrating complex matching closely the predicted molecular mass of a DmsD–peptide 2 combination was seen after DSS treatment (Fig. 4) but a weaker band of even larger mass, possibly indicative of two peptides crosslinked to one DmsD protein, was also observed (Fig. 4). It should be noted, however, that this larger DmsD–peptide complex was only seen with peptide 2 (which contains two lysine side chains) and previous *in vitro* calorimetric analysis identified only one high-affinity peptide binding site per DmsD (Guymer *et al.*, 2009). It is possible that, under the conditions used, a pool of peptide 2–peptide 2 self-crosslinks are being generated that retain a free lysine side chain available for crosslinking to DmsD. Taken altogether, the crosslinking experiments and other published evidence suggest that DmsD dimerization is prevented by signal peptide binding and that only one signal peptide binds per DmsD monomer.

In conclusion, this work adds new knowledge to the function of Tat signal peptides and their relationship with their cognate Tat proofreading chaperones. The *S. enterica* selenate reductase is a useful model system for understanding the biosynthesis of complex metalloenzymes and it has here helped to define the binding epitope for DmsD on a Tat signal peptide. The work also reinforces the tangled relationship between two different biochemical processes carried out by the YnfE signal peptide and DmsD. Clearly, the Tat transport activity of the signal peptide overlaps with its chaperone binding function, with the YnfE L24 side chain implicated in both. This finding may also add to the hypothesis that DmsD may have a role in protein targeting (Kostecki *et al.*, 2010; Kuzniatsova *et al.*, 2016), though there is also evidence against that (Ray *et al.*, 2003). On the other hand, this work has not only identified DmsD residues that may be important for signal peptide recognition but also identified others that are involved in an as yet undefined biochemical process outwith peptide binding.

## Supplementary Data

975 Supplementary File 1
