# Supplementary material for: Biosynthesis of selenate reductase in Salmonella enterica: critical roles for the signal peptide and DmsD
Source: Microbiology (Reading). 2016 Dec 21;162(12):2136–46. doi: 10.1099/mic.0.000381 (PMC5203670; doi:10.1099/mic.0.000381)
Supplement: Supplementary File 1 [file mic-162-2136-s001.pdf]

Biosynthesis of selenate reductase in *Salmonella enterica*: critical roles for the signal peptide and DmsD.

Katherine R. S. Connelly, Calum Stevenson, Holger Kneuper and Frank Sargent

SUPPLEMENTARY INFORMATION

SUPPLEMENTARY INFORMATION

Supplementary Figure S1

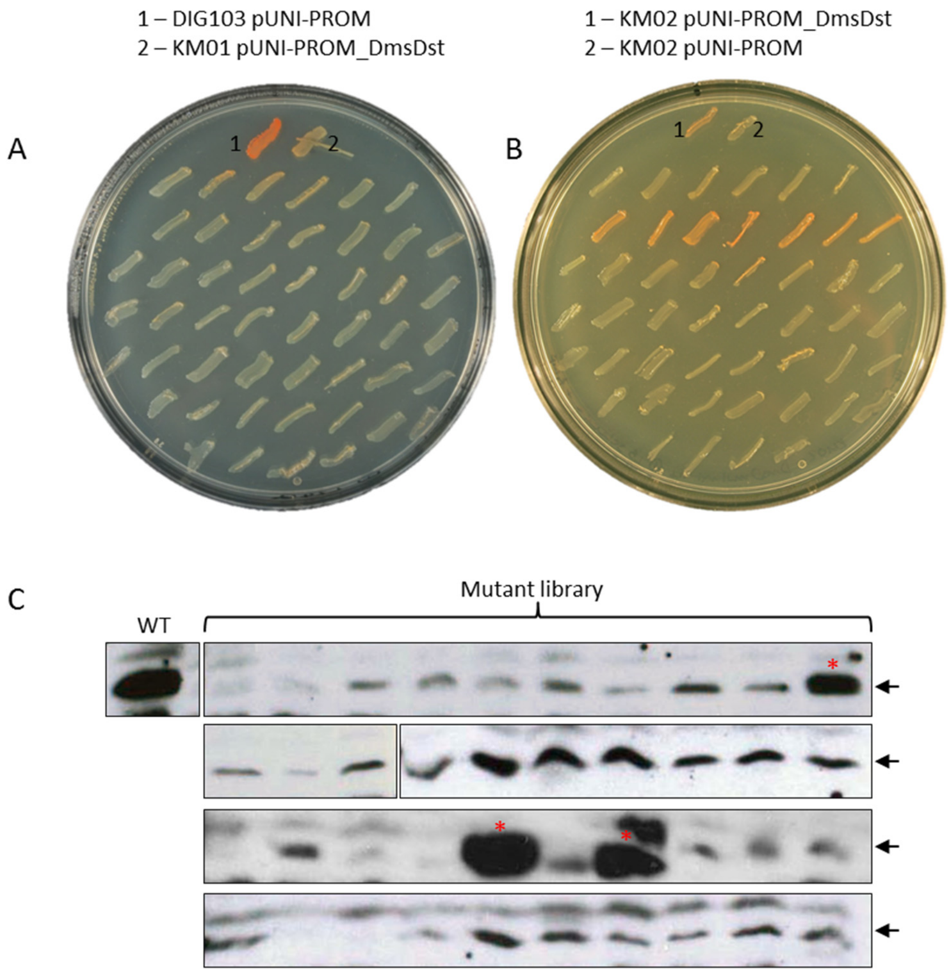

D

```
  1 mttflqrddf avtarvlgal fyyspeshet aplvqallnd dwqaqwplda ealapvaamf
 61 kthseeslpq awqrlfigpy alpsppwgsv wldresvlfg dstlalrqwm rengiqfemq
121 qnepedhfgs llllaawlae ndrhheceql lawhlfpwss rfldvfidha ghpfyqalgq
181 larltlaqwq aqliipvavk plfr
```

Supplementary Figure S1: Identification of amino acid substitutions in DmsD that disrupt selenate reductase biosynthesis. **(A)** Screen of *dmsD* random mutant library in KM01 (*ynfE L24Q*). Cells were transformed with the pUNI-PROM_DmsDst mutant library and plated directly onto LB agar plates containing 10 mM sodium selenate. Positive control (1) was DIG103 pUNI-PROM and negative control (2) was KM01 pUNI-PROM_DmsDst. The presence or absence of selenate reductase activity can be observed through the lack or appearance of red cells. After 36 hours of anaerobic growth, plates were examined and colonies displaying the desired phenotype, for KM01 red cells, were patched onto LB agar + 10 mM sodium selenate plates to confirm the observed phenotype. **(B)** Screen of DmsD random mutant library as in A in KM02 (*ynfE A28Q*). Positive control (1) was KM02 pUNI-PROM_DmsDst and negative control (2) was KM02 pUNI-PROM. **(C)** DmsD variants observed as disrupting selenate reductase activity in KM02 were tested for expression and stability by Western analysis of whole cell samples. Antibodies: Anti-DmsD – 1:20,000. Anti-rabbit – 1:10,000. Individual variants present at similar levels to native DmsD were mini-prepped and sequenced (*). **(D)** Primary amino acid sequence of *S. enterica* DmsD with amino acid residues highlighted that were substituted in DmsD variants found to have reduced selenate reductase activity.

Supplementary Figure S2

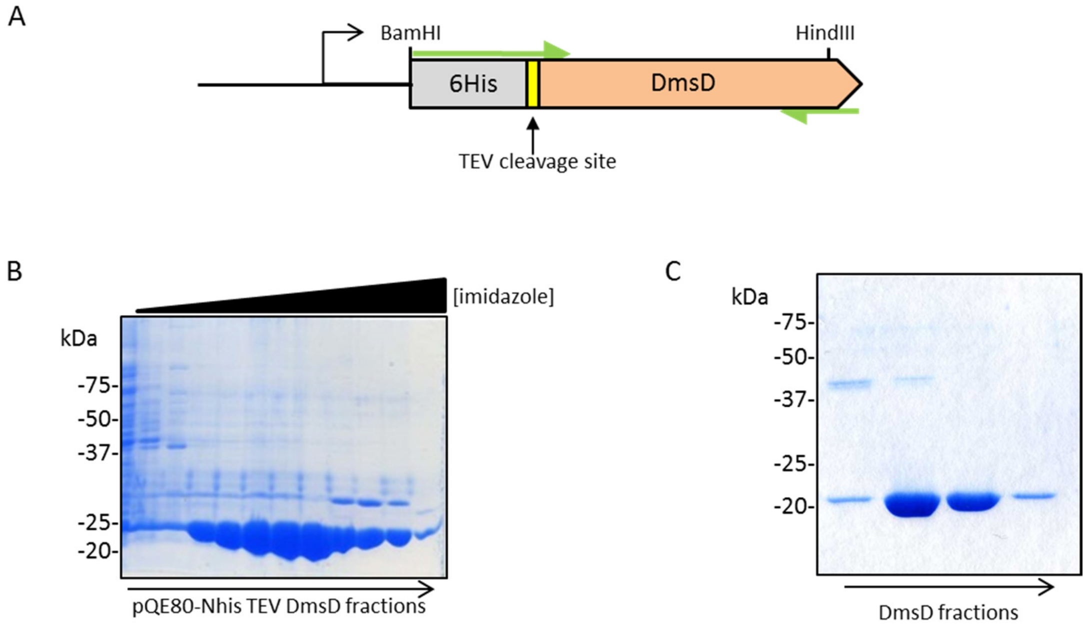

Supplementary Figure S2: Purification of recombinant DmsD.
(A) Construct design for pQE80-NHis TEV DmsD. Primers designed are indicated by green arrows. **(B)** BL21(DE3) pLysS cells overproducing DmsD^{His} were lysed, with the resultant soluble fraction being subjected to Ni-IMAC. Fractions were analysed through SDS-PAGE (12.5 % acrylamide gel), and those containing DmsD^{His} were pooled and incubated overnight in the presence of TEV protease in order to cleave the His-tag. Cleaved DmsD was then isolated by reverse Ni-IMAC. **(C)** DmsD isolated from reverse Ni-IMAC was pooled, concentrated and subjected to further purification by size exclusion chromatography with a Superdex 75 10/300 GL column. Fractions were visualised by SDS-PAGE (12.5 % [w/v] acrylamide gel).

SUPPLEMENTARY FIGURE S3

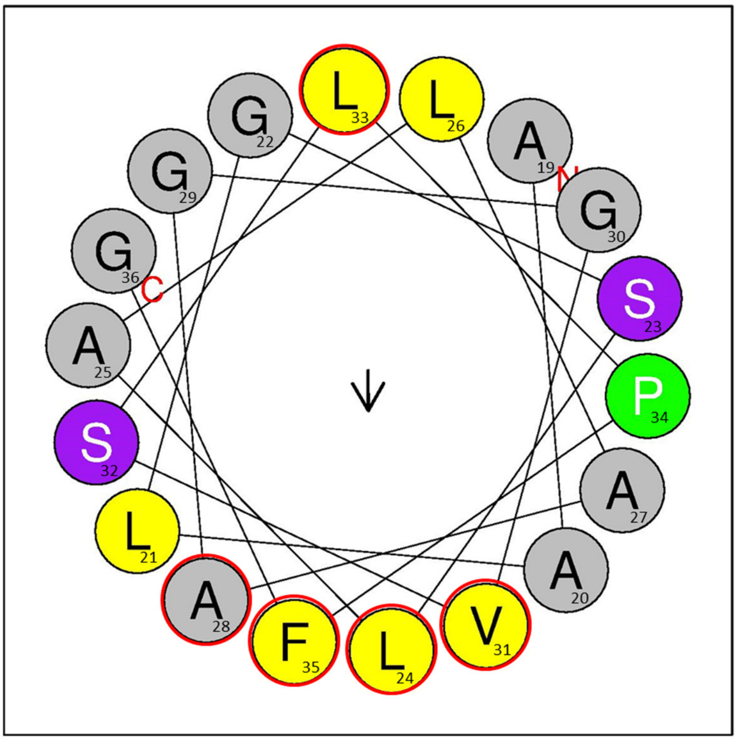

19-AALGSLALAAGGVSLPFG-36

Supplementary Figure S3: Helical wheel modelling of spYnfE.
The spYnfE hydrophobic region displayed as an α-helical wheel projection. Amino acids highlighted as important in DmsD interactions are circled red. Leu-24, Ala-28, Val-31 and Phe-35 form a face on one side of the helix, whilst Leu-33 appears to be localised on the opposite side of the helix. Image assembled using heliQuest (http://heliquest.ipmc.cnrs.fr/cgi-bin/ComputParamsV2.py).
